# A Hematologic Masquerader: Progressive Familial Intrahepatic Cholestasis Type 3 Presenting as Anemia, Hepatosplenomegaly, and Recurrent Bleeding in a Child

**DOI:** 10.1002/ccr3.72961

**Published:** 2026-06-18

**Authors:** Njood Alwadei

**Affiliations:** ^1^ Department of Pediatric Hematology/Oncology King Khalid University Medical City Abha Saudi Arabia

**Keywords:** ABCB4 mutation, esophageal varices, iron deficiency anemia, PFIC‐3, portal hypertension, progressive familial intrahepatic cholestasis type 3

## Abstract

Progressive familial intrahepatic cholestasis type 3 (PFIC‐3), caused by pathogenic variants in the ABCB4 gene, is a rare inherited cholestatic liver disorder that often presents later in childhood. In some patients, hematologic manifestations may dominate the clinical picture and delay recognition of the underlying liver disease. We report a 4‐year‐old girl referred for evaluation of anemia, hepatosplenomegaly, and recurrent bleeding. Initial investigation focused on primary hematologic causes. However, imaging revealed portal hypertension with esophageal varices, and genetic testing identified a homozygous pathogenic ABCB4 variant, confirming the diagnosis of PFIC‐3. This case highlights how chronic cholestatic liver disease may masquerade as a primary hematologic disorder and emphasizes the importance of considering systemic causes in children presenting with anemia, hepatosplenomegaly, and bleeding manifestations. Early recognition of underlying liver disease is essential to facilitate appropriate multidisciplinary management and prevent potentially life‐threatening complications.

## Key Clinical Message

- Chronic cholestatic liver disease can masquerade as primary hematologic pathology in children presenting with anemia and bleeding.
- Iron deficiency anemia associated with hepatosplenomegaly should prompt evaluation for secondary causes, particularly portal hypertension.
- Normal platelet counts and coagulation studies do not exclude clinically significant bleeding in chronic liver disease.
- Pediatric hematologists play a key role in identifying masquerading systemic diseases and enabling timely multidisciplinary care.

## 1 Introduction

Anemia associated with hepatosplenomegaly in children commonly prompts evaluation for primary hematologic disorders, including malignancy, hemolytic anemia, and bone marrow failure syndromes. While iron deficiency anemia is frequently encountered in pediatric practice, the presence of organomegaly and bleeding manifestations should raise concern for secondary systemic causes.

Chronic liver disease is increasingly recognized as a cause of secondary hematologic abnormalities. Portal hypertension may result in chronic occult gastrointestinal blood loss and iron deficiency anemia, even in the absence of overt bleeding [1]. Furthermore, bleeding risk in chronic liver disease is complex and is best explained by the concept of rebalanced hemostasis, in which reductions in procoagulant factors are counterbalanced by decreases in anticoagulant proteins, leading to a fragile but compensated hemostatic equilibrium [2, 3].

Progressive familial intrahepatic cholestasis type 3 (PFIC‐3), caused by pathogenic variants in the ABCB4 gene encoding the MDR3 phospholipid transporter, is a rare inherited cholestatic liver disorder. Defective phospholipid secretion into bile results in toxic detergent‐like bile that injures cholangiocytes and promotes cholestasis, biliary fibrosis, and progressive liver disease. PFIC‐3 often presents later in childhood than PFIC‐1 and PFIC‐2 [4, 5, 6, 7]. In some cases, secondary hematologic manifestations may dominate the clinical picture and obscure the underlying diagnosis.

We report a case of PFIC‐3 initially referred to pediatric hematology for evaluation of anemia, hepatosplenomegaly, and recurrent bleeding, highlighting the diagnostic challenges and the critical role of hematologists in recognizing systemic masqueraders.

## 2 Case History/Examination

A 4‐year‐old girl was referred to the pediatric hematology clinic from a peripheral hospital for evaluation of anemia associated with hepatosplenomegaly.

She had a history of intermittent pallor and recurrent epistaxis, with occasional vomiting but no documented hematemesis. There was no history of jaundice, dark urine, prolonged fever, weight loss, bone pain, or recurrent infections. Oral iron therapy had been prescribed previously but was taken inconsistently for approximately 2 weeks, which was insufficient to assess therapeutic response adequately.

She was born full‐term via normal vaginal delivery with no neonatal complications. Growth and developmental milestones were appropriate for age. The parents were consanguineous.

Family history was notable for a sibling with a similar presentation who died following massive hematemesis secondary to suspected variceal bleeding in the context of chronic liver disease without a definitive diagnosis. Adult paternal relatives were reported to have chronic liver disease and hepatomegaly. This family history, together with parental consanguinity, raised suspicion for an autosomal recessive inherited hepatobiliary disorder.

On examination, the patient appeared well and was not in distress. She was neither pale nor jaundiced. Dysmorphic features were noted, including prognathism and a small triangular face. Abdominal examination revealed hepatosplenomegaly without ascites. Other systemic examinations were unremarkable.

## 3 Differential Diagnosis, Investigations and Treatment

Initial laboratory evaluation showed microcytic hypochromic anemia (hemoglobin 8.8 g/dL, mean corpuscular volume and mean corpuscular hemoglobin were 64 fL and 19 pg., respectively), with normal white blood cell and platelet counts. Serum ferritin was 3.61 μg/L and transferrin saturation was 9.5%, further supporting iron deficiency. Reticulocyte count was mildly elevated at 3.50%. Platelet count was 231 × 10^9^/L. Prothrombin time was 14 s, INR 1.08, activated partial thromboplastin time 39 s and fibrinogen 2 g/L. Lactate dehydrogenase was normal, and direct antiglobulin test was negative. Peripheral blood smear showed no abnormal cells or blasts. Hemoglobin electrophoresis and glucose‐6‐phosphate dehydrogenase levels were normal.

Liver enzymes were mildly elevated (AST 93 U/L, ALT 44.9 U/L, and ALP 200 U/L) with elevated cholestatic markers (GGT 116 U/L), while bilirubin remained within normal limits.

Viral studies showed Epstein–Barr virus IgM positivity; however, the overall clinical picture was not consistent with acute Epstein–Barr virus infection. The patient had a chronic course, no fever, no lymphadenopathy, no atypical lymphocytosis on peripheral smear, and no biochemical pattern suggestive of acute viral hepatitis. In addition, the liver enzyme pattern, with disproportionately elevated cholestatic markers (GGT and ALP) relative to transaminases, was more consistent with chronic cholestasis than acute viral hepatitis. Therefore, this finding was considered incidental and unlikely to explain the presentation.

Given the unexplained anemia, bleeding manifestations, dysmorphic features, and hepatosplenomegaly, extended hematologic evaluation was undertaken. Bone marrow aspiration and biopsy revealed normocellular marrow with preserved trilineage hematopoiesis and no evidence of malignancy or marrow failure.

Abdominal ultrasonography demonstrated hepatosplenomegaly with features suggestive of portal hypertension. Upper gastrointestinal endoscopy revealed esophageal varices without red signs, and band ligation was performed.

Whole‐exome sequencing identified a homozygous pathogenic ABCB4 variant, NM_018849.2:c.2906G>A p.(Arg969His), confirming progressive familial intrahepatic cholestasis type 3 (PFIC‐3).

The chronological diagnostic course is summarized in Table 1. The differential diagnoses considered during evaluation are summarized in Table 2.

**TABLE 1:** Chronological clinical timeline.

Time point	Clinical findings	Investigations	Interpretation/Clinical action
Initial presentation	Pallor, recurrent epistaxis, hepatosplenomegaly	Hb 8.8 g/dL, MCV 64 fL	Microcytic anemia identified
Initial laboratory workup	No jaundice, normal platelets	Iron studies consistent with iron deficiency	Nutritional iron deficiency suspected
Expanded hematologic evaluation	Dysmorphic features, parental consanguinity, family history of fatal bleeding	Bone marrow aspiration and biopsy: normocellular marrow with preserved trilineage hematopoiesis	Primary hematologic malignancy and marrow failure excluded
Hepatobiliary assessment	Persistent hepatosplenomegaly	Abdominal ultrasound suggestive of portal hypertension	Secondary systemic cause suspected
Endoscopic evaluation	Recurrent epistaxis and concern for GI bleeding	Esophageal varices identified; band ligation performed	Portal hypertension confirmed
Genetic testing	Strong suspicion of inherited disorder	Whole‐exome sequencing: homozygous pathogenic ABCB4 variant	Diagnosis of PFIC‐3 established
Follow‐up	Clinical stabilization	Hemoglobin improved with iron supplementation and variceal management	Anemia attributed to chronic blood loss secondary to portal hypertension

**TABLE 2:** Differential diagnosis considered during evaluation.

Diagnostic category	Considered	Basis for consideration	Reason for Exclusion/Confirmation
Hematologic malignancy	Yes	Anemia + hepatosplenomegaly	Normal marrow morphology; no blasts
Bone marrow failure syndromes	Yes	Cytopenia with organomegaly	Preserved trilineage hematopoiesis
Hemolytic anemia	Yes	Elevated reticulocyte count	Normal LDH; negative DAT; no hemolytic features
Nutritional iron deficiency anemia	Yes	Microcytic anemia	Present but insufficient to explain organomegaly, recurrent bleeding, or the significant family history.
Chronic liver disease with portal hypertension	Yes	Organomegaly + bleeding	Confirmed by imaging and endoscopy
Inherited cholestatic liver disorder (PFIC‐3)	Yes	Consanguinity + family history	Confirmed by pathogenic ABCB4 mutation
Lysosomal storage disorders (e.g., Gaucher disease)	Yes	Hepatosplenomegaly + anemia	No supportive marrow findings; definitive ABCB4 pathogenic variant identified

## 4 Conclusion and Results (Outcome and Follow‐Up)

From a hematologic perspective, the anemia was attributed to secondary iron deficiency resulting from chronic occult blood loss related to portal hypertension rather than a primary hematologic disorder.

Following endoscopic management and iron supplementation, the patient demonstrated clinical stabilization. Hemoglobin levels improved with appropriate iron therapy. She was transitioned to hepatology and gastroenterology services for ongoing management of portal hypertension and chronic liver disease. Following confirmation of the diagnosis, ursodeoxycholic acid therapy was initiated under gastroenterology supervision as first‐line medical management for PFIC‐3. Ursodeoxycholic acid was initiated recently; however, the first post‐treatment follow‐up assessment was still pending at the time of manuscript revision.

Hematology follow‐up was continued as needed, with no evidence of primary hematologic pathology during subsequent evaluations.

## 5 Discussion

Anemia associated with hepatosplenomegaly is a common indication for referral to pediatric hematology and often raises concern for primary hematologic disorders such as malignancy, hemolytic anemia, or bone marrow failure syndromes. While microcytic hypochromic anemia is frequently attributed to nutritional iron deficiency in young children, secondary causes related to chronic liver disease may masquerade as primary hematologic pathology and lead to diagnostic delay if the broader clinical context is not considered.

Although iron deficiency anemia was initially identified in this patient, several features were inconsistent with an isolated nutritional etiology, including hepatosplenomegaly, recurrent bleeding manifestations, dysmorphic features, parental consanguinity, and a family history of fatal gastrointestinal bleeding. These red flags warranted a broader diagnostic approach. Comprehensive hematologic evaluation, including bone marrow examination, was essential not only to exclude malignancy and marrow failure but also to confidently redirect diagnostic reasoning toward a systemic cause.

Chronic liver disease is increasingly recognized as a cause of secondary hematologic abnormalities. Portal hypertension can result in chronic occult gastrointestinal blood loss through portal hypertensive gastropathy and mucosal congestion, leading to iron deficiency anemia even in the absence of overt gastrointestinal bleeding [1]. In pediatric patients, this mechanism may be overlooked, particularly when anemia is mild to moderate or shows a partial response to iron supplementation.

Bleeding manifestations in chronic liver disease are complex and are best explained by the concept of rebalanced hemostasis. In this model, reductions in procoagulant factors are counterbalanced by parallel decreases in anticoagulant proteins, resulting in a fragile but functionally compensated hemostatic state [2, 3]. Consequently, conventional coagulation tests such as PT, INR, aPTT, and platelet count do not reliably predict bleeding risk, and clinical decisions should not be based on these parameters alone. Clinically significant bleeding may occur despite apparently reassuring laboratory parameters, as illustrated in this patient and her affected sibling.

In children with portal hypertension, bleeding is often driven by local vascular pathology rather than systemic coagulation defects. Esophageal varices, portal hypertensive gastropathy, and congested nasal and gastrointestinal mucosa are common sources of bleeding in this population [1, 8]. Moreover, splenomegaly associated with portal hypertension may contribute to platelet sequestration and reduced effective circulating platelet mass, which can further aggravate bleeding tendency even when bone marrow production is preserved [8]. This explains the recurrent epistaxis observed in the present patient and the fatal variceal hemorrhage in the affected sibling, despite the absence of a primary coagulation disorder. In addition, impaired absorption of fat‐soluble vitamins in chronic cholestatic liver disease, particularly vitamin K deficiency, may further increase bleeding tendency [2, 3].

Progressive familial intrahepatic cholestasis type 3, caused by pathogenic variants in the ABCB4 gene, represents a particular diagnostic challenge. Unlike other PFIC subtypes, PFIC‐3 may present later in childhood with less prominent early cholestasis, allowing extrahepatic manifestations such as anemia and bleeding to dominate the clinical picture [4, 5, 6, 7]. The disease may progress to fibrosis, cirrhosis, portal hypertension, and end‐stage liver disease, with liver transplantation remaining the definitive treatment when medical therapy fails [4, 5, 6, 7]. The presence of parental consanguinity, dysmorphic features, and a strong family history supports the use of genomic testing in this case.

Although family history was an important diagnostic clue in this case, clinicians should recognize that inherited cholestatic liver disorders may also occur in the absence of a clear family history. Therefore, these conditions should remain in the differential diagnosis of children presenting with unexplained hepatosplenomegaly, anemia, or recurrent bleeding.

The dysmorphic features observed in this patient, including prognathism and triangular facial appearance, are not recognized manifestations of ABCB4‐related disease. Whole‐exome sequencing identified no additional definitively pathogenic variants explaining these findings. However, a heterozygous variant of uncertain significance in HNRNPH1, a gene associated with neurodevelopmental disorders that may include craniofacial dysmorphism, was also reported. Given its uncertain classification and the absence of clear neurodevelopmental abnormalities in this patient, its clinical relevance remains unclear. Therefore, the dysmorphic features may represent nonspecific phenotypic characteristics rather than a confirmed syndromic association.

Whole‐exome sequencing provided the diagnostic turning point by identifying a homozygous pathogenic ABCB4 variant, unifying the patient's clinical features and family history under a single diagnosis. Genomic testing has been shown to significantly improve diagnostic yield in children with unexplained multisystem disease, particularly in populations with high rates of consanguinity [9]. Following confirmation of the diagnosis, the family was counseled regarding genetic implications, and cascade family screening was recommended.

This case underscores the critical role of pediatric hematologists in recognizing when anemia and bleeding are secondary manifestations of systemic disease. Early identification of inherited cholestatic liver disorders may prevent life‐threatening complications and highlights the importance of multidisciplinary collaboration in complex pediatric presentations.

A limitation of this case is the lack of access to advanced diagnostic tests, including serum bile acids, fat‐soluble vitamin levels, and comprehensive platelet function studies (e.g., platelet aggregation assays). This reflects local resource constraints. Nevertheless, the diagnosis and clinical decisions were supported by consistent clinical findings and available laboratory data, and additional testing was unlikely to alter the management course.

## Author Contributions

**Njood Alwadei:** conceptualization, data curation, investigation, supervision, writing – original draft, writing – review and editing.

## Funding

The author has nothing to report.

## Ethics Statement

The author has nothing to report.

## Consent

Written informed consent was obtained from the patient's legal guardians for publication of this case report and accompanying clinical information. Identifying details have been anonymized to protect patient privacy.

## Data Availability

The data supporting the findings of this study are available from the corresponding author upon reasonable request.
